# Prevalence, Characteristics, and Management of Pediatric Ocular Trauma in Riyadh, Saudi Arabia: A Retrospective Analysis

**DOI:** 10.3390/healthcare12161673

**Published:** 2024-08-22

**Authors:** Balsam Alabdulkader, Yara Alsiwat, Hessa Almatar, Bayan Albdah, Ali Almustanyir, Essam Almutleb, Norah Alkanhal, Ahmed Almazroa

**Affiliations:** 1 Department of Optometry, College of Applied Medical Sciences, King Saud University, Riyadh 11451, Saudi Arabia; alabdulkader@ksu.edu.sa (B.A.); yaraalsiwat@gmail.com (Y.A.); aalmustanyir@ksu.edu.sa (A.A.); esalsarhani@ksu.edu.sa (E.A.); 2 AI and Bioinformatics Department, King Abdullah International Medical Research Center, Riyadh 11481, Saudi Arabia; almatarh@kaimrc.edu.sa; 3 King Saud Bin Abdulaziz University for Health Sciences, Riyadh 11481, Saudi Arabianhkanhal@cdc.gov.sa (N.A.); 4 Department of Biostatistics and Bioinformatics, King Abdullah International Medical Research Center, Riyadh 11481, Saudi Arabia

**Keywords:** eye injuries, children, Birmingham Eye Trauma Terminology, closed globe injuries, open globe injuries

## Abstract

Purpose: Ocular trauma is a major cause of visual impairment; however, little is known about its burden in Saudi Arabia. Therefore, this study aimed to determine the epidemiological characteristics of ocular trauma in pediatric patients in Riyadh, Saudi Arabia. Patients and methods: Medical records of pediatric patients diagnosed with eye injuries between January 2016 and December 2020 were retrospectively reviewed. Demographic and injury characteristics were collected, and ocular trauma injuries were classified according to the Birmingham Eye Trauma Terminology. Results: A total of 855 injured patients were included in the study, of whom 525 (61.4%) were boys. Patient age ranged from one month to 18 years. Most ocular injuries occurred in children aged 5–9 years. The injuries were more prevalent in boys than in girls. Closed globe injuries accounted for 70% of cases, open globe injuries for 21%, and other injuries for 9%. Most ocular injuries occurred at home (n = 87, 42%), followed by school (n = 61, 30%). Conclusions: These results may inform the implementation and targeting of interventions to reduce or prevent eye injuries in children. Further, they highlight the importance of well-planned prevention programs to prevent eye injuries from occurring in children’s daily lives.

## 1. Introduction

Ocular injuries are one of the leading causes of avoidable blindness in children [1]. Ocular trauma refers to direct physical injury to the eye and its surrounding structures, including the adjacent skin and bone [2]. In 2000, there were 8.9 hospitalizations for pediatric eye injuries per 100,000 people aged ≤ 20 years in the US [3]. According to several studies, the prevalence of severe vision impairment or blindness in children due to ocular trauma varies between 2 and 14% globally [4,5], with open globe traumas accounting for 25% of these incidents [6]. Such accidents are a causative factor in the development of monocular blindness and can result in substantial disability, having profound psychological and social consequences in affected children [1]. Children have a natural curiosity, are prone to mimicking without considering the consequences, and more likely to sustain eye injuries due to their underdeveloped motor skills and lack of common sense. Although they are supposed to have limited access to dangerous situations and potentially harmful objects, childhood eye injuries are a typical occurrence, accounting for approximately 30% of all injuries [7]. Therefore, a closer examination of the causes and consequences of eye injuries in children is necessary in light of the variations in demographics and etiology, challenges in initial evaluation, requirement for extended follow-up, and possibility of amblyopia [7].

Moreover, while not typically fatal, the severity of ocular trauma can range from minor to serious medical emergencies that may result in permanent eye damage and visual impairment [8]. Ocular trauma is a significant global public health concern, with increasing interest in its prevention, especially in the pediatric population. Moreover, approximately 90% of ocular traumas can be prevented [9] by implementing preventive measures and using protective eyewear. However, current strategies require more effective implementation. Thus, a thorough and methodical examination of children who have experienced ocular trauma will help reduce morbidity and improve long-term visual outcomes.

Several risk factors, including sex, age, socioeconomic status, and location play significant roles in the prevalence of ocular trauma among children. Boys are more likely to suffer from eye injuries than girls [10,11,12,13,14], and young individuals are more prone to eye injuries than older individuals [10,13,14]. Furthermore, developing countries with limited access to primary care have a higher prevalence of ocular trauma than developed countries [11,15,16], and children residing in industrial regions may have a higher prevalence than those residing in other regions [17].

Furthermore, a few studies related to ocular trauma in children conducted in Saudi Arabia [18,19,20,21,22] revealed that in a majority of the optic nerve avulsion cases, permanent vision impairment occurred, and metallic object injuries were the most prevalent cause of optic nerve avulsion [18]. Boys mainly suffered fireworks-related eye injuries [20], and visual outcomes of chemical burns in children were not promising even after one year of treatment [19]. Pointed door handles also posed a significant risk of ocular and periocular injuries among young children [22]. Moreover, primary care physicians working at primary care centers in Riyadh, Saudi Arabia, had good knowledge about eye trauma among children aged < 14 years [21]. The knowledge level was significantly associated with the physicians’ age, experience, history of dealing with a child with eye trauma, and history with difficult eye trauma cases.

The Birmingham Eye Trauma Terminology (BETT) [23] is an internationally standardized system developed by Kuhn et al. that allows an accurate description of all types of eye injuries, improving communication between eye care providers. The BETT classification has been used in numerous studies to describe ocular trauma types [6,24,25,26,27].

The epidemiology of ocular trauma has been described in several countries [13,14,28,29]; however, its prevalence, risk factors, and outcomes in Saudi Arabia have received little attention. According to a previous study at a tertiary eye hospital in Riyadh, Saudi Arabia, many children suffering from open globe injuries were unable to achieve normal functional vision despite the intervention of skilled care providers [30]. This underscores the need for further studies to understand the prevalence and characteristics of ocular trauma in children in Saudi Arabia and to develop prevention measures and raise public awareness.

Therefore, this study aimed to evaluate the epidemiology of eye injuries in children in Riyadh, Saudi Arabia, from 2016 to 2020. The objectives of this study were to determine the epidemiology of ocular trauma, define its clinical characteristics, and provide information on the causes of pediatric open and closed globe injuries in a major pediatric facility. This study will provide insights into pediatric ocular trauma in Riyadh and inform prevention and treatment strategies.

## 2. Materials and Methods

### 2.1. Study Design

**Figure 1:**
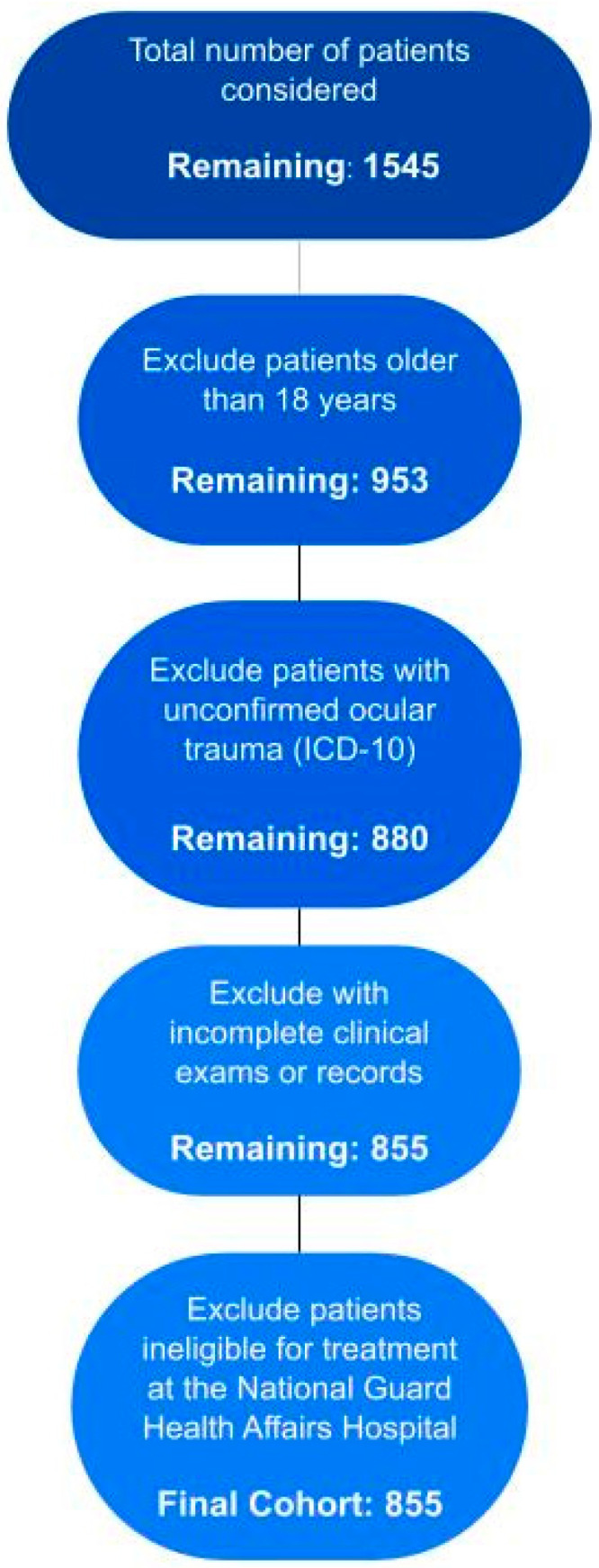
Patient selection flowchart.

This cross-sectional, retrospective, observational study was conducted using data from the Trauma Registry of King Abdulaziz Medical City, Riyadh, Saudi Arabia, at the National Guard Health Affairs Hospital. Data from patients with ocular trauma who visited the hospital between January 2016 and December 2020 were extracted from the Trauma Registry. The inclusion criterion was a confirmed diagnosis of ocular trauma with a registered International Classification of Diseases, Tenth Revision code [31]. Patients with incomplete clinical examinations or medical records because of referral to another hospital or who were ineligible for treatment at the National Guard Health Affairs Hospital were excluded (Figure 1). During each visit, the patients’ background characteristics, including demographic information, previous ocular history, injury location and extent, and cause of ocular trauma, were recorded. Ocular traumas were classified according to the BETT [28], as follows: closed globe injuries (contusion and lamellar laceration) and open globe injuries (penetration, perforation, intraocular foreign body injury, and rupture).

The study protocol complied with the principles of the Declaration of Helsinki and was approved by the King Abdullah International Medical Research Center Research Ethics Committee (approval number: RC20/677/R; 21 January 2021). The requirement for informed consent was waived due to the retrospective design of the study.

### 2.2. Data Analysis

Data were analyzed using SAS software (version 9.4; SAS Institute, Cary, NC, USA). Descriptive statistics were used to characterize the type of trauma and the sociodemographic characteristics of the patients. Injuries were classified as open and closed globe injuries according to the BETT [23], while other types of injuries were classified as “other”. Chi-square analysis was performed to evaluate the associations between the type of injury and age group and sex. Statistical significance was set at *p* < 0.05.

## 3. Results

### 3.1. Sociodemographic Characteristics

A total of 855 pediatric ocular trauma cases were retrospectively reviewed during the study period. Patient age ranged from one month to 18 years, with a mean age of 6.4 ± 3.8 years. Among all the patients, 525 (61.4%) were boys and 330 (38.6%) were girls, yielding a male-to-female ratio of 1.6:1. The study population was divided into four age groups: 0–4 years (31.5%, n = 269), 5–9 years (45.8%, n = 392), 10–14 years (18.7%, n = 160), and 15–18 years (4%, n = 34). The right and left eyes were affected in 405 (47.4%) and 411 (48%) patients, respectively, whereas the affected eye was not specified in 39 (4.6%) patients. No significant differences were observed between right and left eye injuries according to age (*p* = 0.811). Most injuries occurred in patients aged 5–9 years (46%, n = 378), followed by those aged 0–4 years (31%, n = 260), 10–14 years (19%, n = 157), and 15–18 years (4%, n = 34).

### 3.2. Ocular Injury Observations

**Figure 2:**
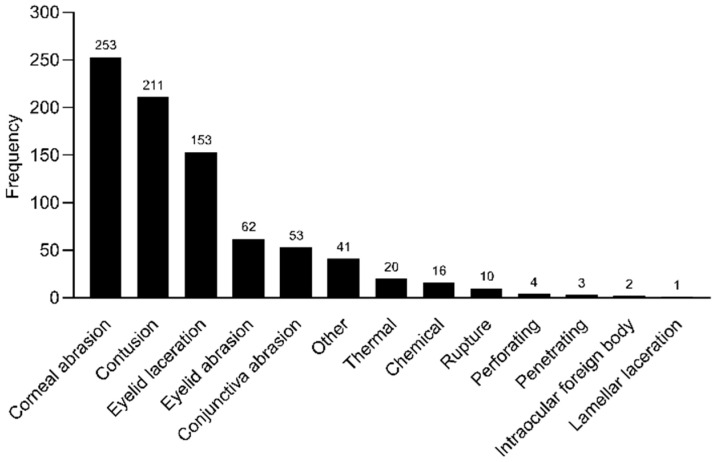
Injury types for children from 2016 to 2020 (n = 855).

The most common type of injury was corneal abrasion (30.5%, n = 253), followed by contusion (25.4%, n = 211). Lamellar laceration, defined as a partial-thickness wound to the eye wall, was the least common type of injury (0.12%, n = 1). The different injuries encountered by the patients are illustrated in Figure 2.

Closed globe injuries were the most common type (70%), followed by open globe injuries (21%) and other types of injuries (9%). There was a significant difference in the type of injury among different age groups and between boys and girls, with children aged 5–9 years having more injuries than other age groups (*p* < 0.001) (Table 1), and boys having more injuries than girls (*p* < 0.001) (Table 2).

**Figure 3:**
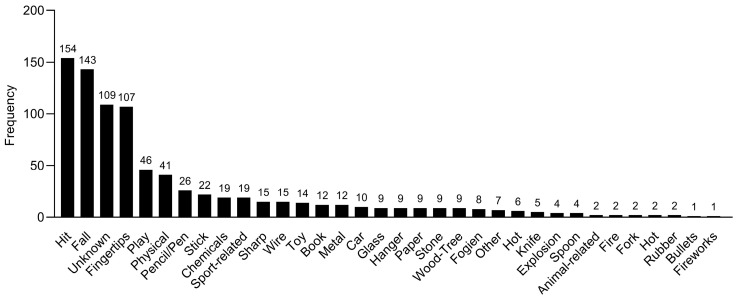
Causes of ocular trauma from 2016 to 2020 (n = 855).

Various causes of ocular trauma were identified, and the most common cause was being hit by an object or body part (18%, n = 154), followed by falling (16.7%, n = 143). The other causes of ocular trauma that occurred during the study period are shown in Figure 3.

**Figure 4:**
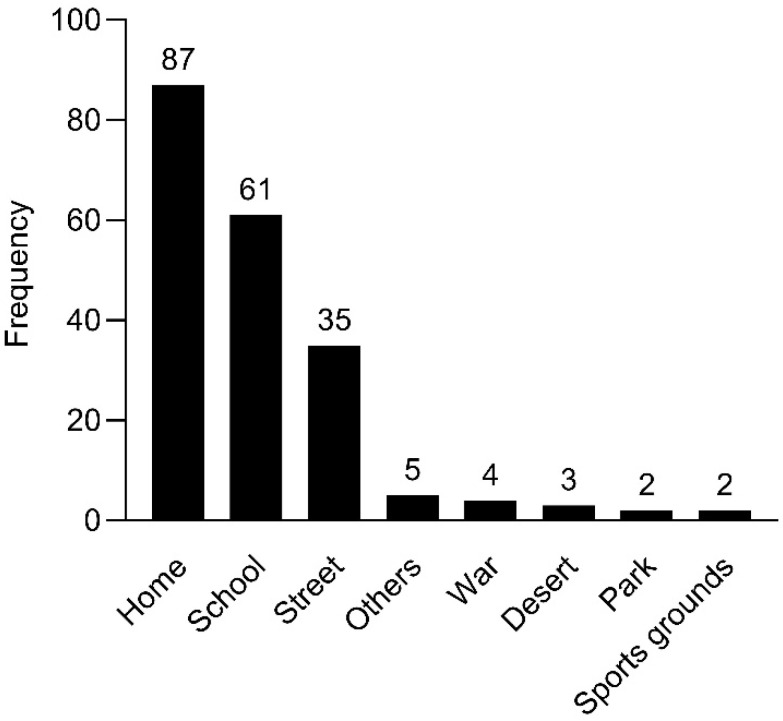
Location of ocular trauma occurrence from 2016 to 2020 (n = 199).

Most ocular injuries occurred at home (43.7%, n = 87), followed by school (30.6%, n = 61). The locations of ocular injuries are presented in Figure 4.

## 4. Discussion

Ocular trauma is a major cause of monocular blindness worldwide. However, limited information is available on its epidemiology in Saudi Arabia. Therefore, this study aimed to determine and summarize the epidemiological and clinical characteristics of ocular trauma in children in Saudi Arabia. The results indicated that ocular trauma was predominantly more common in boys, who accounted for 61.4% of the cases, compared to girls. This gender disparity is aligned with findings from similar studies conducted internationally, where boys typically exhibit higher rates of ocular injuries [1,20,32,33]. This trend can be attributed to several factors: boys are often more involved in physically vigorous and aggressive activities, exhibit higher risk-taking behaviors, and may experience different levels of supervision and cultural expectations. Most ocular traumas were uncomplicated, with corneal abrasion and contusion being the most common types of injury. Our results also showed that 46% of ocular injuries occurred in children aged 5–9 years old. The prevalence of ocular trauma among children aged 5–9 years may be attributed to developmental, behavioral, and environmental factors, with a significant proportion of these injuries occurring at home. This age group is marked by increased independence and physical activity, yet their coordination and judgment skills are still maturing. As children engage more robustly in play and exploratory behaviors within the home environment—where many assume safety and thus may provide less stringent supervision—they encounter risks from everyday objects and activities. This situation is compounded by their natural curiosity and a lack of awareness about potential dangers, making domestic settings a common backdrop for these incidents. Enhanced parental supervision, along with targeted safety education at home, can help mitigate these risks.

Moreover, ocular trauma is responsible for 7% of trauma hospitalizations and 10–15% of presentations for ophthalmic reasons. Patients aged < 18 years account for more than a third of the occurrences of ocular trauma, and one in five cases occur before the age of 12 years [6]. The most common group of people exposed to high-risk ocular trauma that results in vision impairment is young children (less than five years old) [34]. Furthermore, between 2 and 14% of cases of vision impairment or blindness worldwide are attributed to ocular trauma [4,5,35].

The current study primarily observed the highest incidence in younger children, with 46% of injuries occurring in those aged 5–9 years. This contrasts with findings from Puodziuviene et al. [1], where 72.8% of injuries occurred in children over 7 years of age, and Shoja and Miratashi [36], who reported the majority (58.3%) of injuries in the 7–12-year age group. It is important to note that while the specific age ranges differ slightly, there is a significant overlap between the groups, particularly in the 7–9-year range, which is included in both the highest incidence group in our study and the age groups noted in the other studies. This overlap suggests that while our findings show a broader distribution of ages, particularly at younger ages, the core age group at highest risk (7–9 years) is consistently identified across studies. Possible reasons for the broader age distribution in our study could include regional variations, differing safety measures, or activity patterns among younger children. Nonetheless, depending on age and causes of ocular damage, the male preponderance is rather consistent, with the male–female ratio ranging from 2/1 to 9/1 [34,35,37,38,39]. Patients with ocular trauma often vary in age from 6 to 11 years [6,37,38].

Consistent with other studies, the home was found to be the leading location for eye injuries [1]. This may be because young children spend more time at home than older children. Additionally, the data included ocular injuries from 2020, when many countries were under lockdown due to the coronavirus disease 2019 (COVID-19) pandemic, and people were required to stay home. A study conducted in Paris, France, which compared the use of eye-related emergency services by children before the COVID-19 pandemic and during the lockdown imposed due to the COVID-19 pandemic [40], revealed that the use of emergency services decreased by 46% during the lockdown. Additionally, visits due to benign causes and ocular trauma also decreased; however, visits for more severe pathologies remained unaffected. This decrease encompassed visits for ocular trauma, despite an increase in time spent at home. The apparent contradiction might be explained by several factors: a reduction in riskier activities such as sports, altered healthcare-seeking behavior, with families avoiding hospital visits for minor injuries due to infection fears, potential underreporting during the pandemic, and a focus on more severe conditions during emergency visits. These factors suggest that the lockdown’s impact on ocular injury patterns is complex, necessitating further research to fully understand these relationships.

Our results showed that closed globe injuries were the most common (70%), followed by open globe injuries (21%) and other types (9%). These results are consistent with those of previous studies [1,5,36,41]. However, Shoja and Miratashi [36] reported a higher average of open globe injuries than closed globe injuries (51.7% vs. 33%). The main reason for this discrepancy may be the differences in the inclusion criteria, as their study did not include outpatient children with ocular injuries. Additionally, Kyei et al. [42] revealed that patients with open globe injuries were approximately 10 times more likely to develop blindness than those with closed globe injuries after adjusting for age and sex; this difference was statistically significant.

The main cause of ocular injury in our study was the impact of an object. Puodziuviene et al. [1] found that blunt objects were the main cause of injuries in children aged 7–12 years and that falls were the second most common cause. Conversely, Lee et al. [5] reported falls to be the leading cause of ocular injuries. Generally, children are more prone to injuries owing to their immature motor skills, limited common sense, and natural curiosity.

The Saudi Ministry of Health has recently developed several initiatives and programs to improve eye care services. One such program [43] focuses on the prevention of blindness and could benefit from the results of this study because ocular trauma is one of the leading causes of blindness. Therefore, understanding the epidemiology of eye trauma is essential for identifying the associated risk factors and developing strategies to reduce and prevent them. Our findings highlight the necessity for healthcare practitioners to be especially attentive in diagnosing and treating ocular injuries in children, particularly within the high-risk 5–9-year age group. An awareness of the common settings for these injuries, primarily the home, allows healthcare practitioners to offer specific preventative advice during routine visits. Additionally, the data support the importance of advocating for protective measures in child-centric environments. Accurate data reporting is crucial for refining these practices and for enhancing the quality of trauma registries, which in turn can guide future studies and improve overall outcomes in pediatric ocular trauma care. The results of our study can contribute to the development of eye health awareness strategies aimed at reducing ocular trauma and preventing blindness while also aiding in the planning of rehabilitation services.

This study utilizes data from one of the few trauma registries in the country, located at King Abdulaziz Medical City, Riyadh. While this represents a significant strength in terms of data reliability and depth, it is also recognized as a limitation since the data are derived from a single center. This may not fully represent the broader national context. Some data were missing owing to incomplete details in the medical records, which may have affected the results. Additionally, our data analysis did not include advanced statistical analyses; therefore, some meaningful insights may remain unknown.

## 5. Conclusions

In conclusion, severe ocular injuries can result in visual impairment and disability, potentially affecting the academic and professional capabilities of children. Children are at greater risk of ocular injuries due to their high levels of physical activity. Thus, our results highlight the importance of adult supervision in providing a safe environment to prevent ocular injuries.

## Figures and Tables

**Table 1:** Injuries stratified by age group (n = 829).

Age Group (Years)	Closed Globe Injuries, Number (%)	Open Globe Injuries, Number (%)	Other Injuries, Number (%)	Total Number of Injuries
0–4	142 (24.48%)	85 (49.42%)	33 (42.86%)	260
5–9	296 (51.03%)	54 (31.40%)	28 (36.36%)	378
10–14	122 (21.03%)	23 (13.37%)	12 (15.58%)	157
15–18	20 (3.45%)	10 (5.81%)	4 (5.19%)	34
Total	580	172	77	829

**Table 2:** Injuries stratified by sex (n = 752).

Sex	Closed Globe Injuries, Number (%)	Open Globe Injuries, Number (%)	Total Number of Injuries
Girls	238 (41.03%)	46 (26.74%)	284
Boys	342 (58.97%)	126 (73.26%)	468
Total	580	172	752

## Data Availability

All data supporting the findings of this study are available within the manuscript.

## Abbreviations

BETT	Birmingham Eye Trauma Terminology
